# Artemisinin-Derived Dimers Have Greatly Improved Anti-Cytomegalovirus Activity Compared to Artemisinin Monomers

**DOI:** 10.1371/journal.pone.0010370

**Published:** 2010-04-28

**Authors:** Ravit Arav-Boger, Ran He, Chuang-Jiun Chiou, Jianyong Liu, Lauren Woodard, Andrew Rosenthal, Lorraine Jones-Brando, Michael Forman, Gary Posner

**Affiliations:** 1 Department of Pediatrics, Johns Hopkins University School of Medicine, Baltimore, Maryland, United States of America; 2 The Sidney Kimmel Comprehensive Cancer Center, Johns Hopkins University School of Medicine, Baltimore, Maryland, United States of America; 3 Department of Chemistry, School of Arts and Sciences, The Johns Hopkins University, Baltimore, Maryland, United States of America; 4 Department of Pathology, Johns Hopkins Medical Institutions, Baltimore, Maryland, United States of America; Saint Louis University, United States of America

## Abstract

**Background:**

Artesunate, an artemisinin-derived monomer, was reported to inhibit Cytomegalovirus (CMV) replication. We aimed to compare the *in-vitro* anti-CMV activity of several artemisinin-derived monomers and newly synthesized artemisinin dimers.

**Methods:**

Four artemisinin monomers and two novel artemisinin-derived dimers were tested for anti-CMV activity in human fibroblasts infected with luciferase-tagged highly–passaged laboratory adapted strain (Towne), and a clinical CMV isolate. Compounds were evaluated for CMV inhibition and cytotoxicity.

**Results:**

Artemisinin dimers effectively inhibited CMV replication in human foreskin fibroblasts and human embryonic lung fibroblasts (EC_50_ for dimer sulfone carbamate and dimer primary alcohol 0.06±0.00 µM and 0.15±0.02 µM respectively, in human foreskin fibroblasts) with no cytotxicity at concentrations required for complete CMV inhibition. All four artemisinin monomers (artemisinin, artesunate, artemether and artefanilide) shared a similar degree of CMV inhibition amongst themselves (in µM concentrations) which was significantly less than the inhibition achieved with artemisinin dimers (P<0.0001). Similar to monomers, inhibition of CMV with artemisinin dimers appeared early in the virus life cycle as reflected by decreased expression of the immediate early (IE1) protein.

**Conclusions:**

Artemisinin dimers are potent and non-cytotoxic inhibitors of CMV replication. These compounds should be studied as potential therapeutic agents for the treatment of CMV infection in humans.

## Introduction

Infection with CMV is common in humans, and is usually asymptomatic [1], [2]. In immunocompromised hosts such as transplant recipients and patients with AIDS, CMV infection is associated with significant morbidity and mortality [3], [4]. It is also the most common congenitally-acquired infection and the leading infectious agent causing mental retardation and deafness in congenitally infected children [5]. In recent years CMV has been associated with a variety of syndromes including hypertension, severe pulmonary complications in patients in intensive care-units, and with a specific brain tumor, glioblastoma multiforme [6]–[9]. Although the exact role of CMV in these syndromes is unclear, CMV replication appears to affect the natural history and outcome of disease processes in immunocompetent individuals as well. Thus, it is important and necessary to develop preventive and treatment modalities for CMV. Despite significant ongoing research effort, there is still no CMV vaccine approved for universal or targeted use.

Available anti-CMV drugs, ganciclovir (GCV), cidofovir and foscarnet, effectively inhibit virus replication by targeting the viral DNA polymerase [10]–[12]. However, use of these drugs is associated with considerable side effects such as bone marrow toxicity (GCV) and nephrotoxicity (foscarnet and cidofovir) [13], [14]. Oral valganciclovir has good bioavailability and is used in bone marrow and organ transplant recipients for CMV prophylaxis and treatment. Valganciclovir has not been approved yet for the treatment of infants with congenital CMV infection; a phase III clinical trial comparing six weeks to six months of valganciclovir therapy is actively enrolling infants. Preliminary data from this trial reveal that GCV-resistant variants emerge during therapy. Drug resistance also develops during prolonged or repeated treatment in the transplant population [15]. Because of the problems associated with currently available anti-CMV compounds, the very limited treatment options for congenital CMV infection and the growing indications for CMV treatment, we urgently need new anti-CMV compounds, especially compounds with high oral bioavailability, low toxicity and low cost.

**Figure 1:**
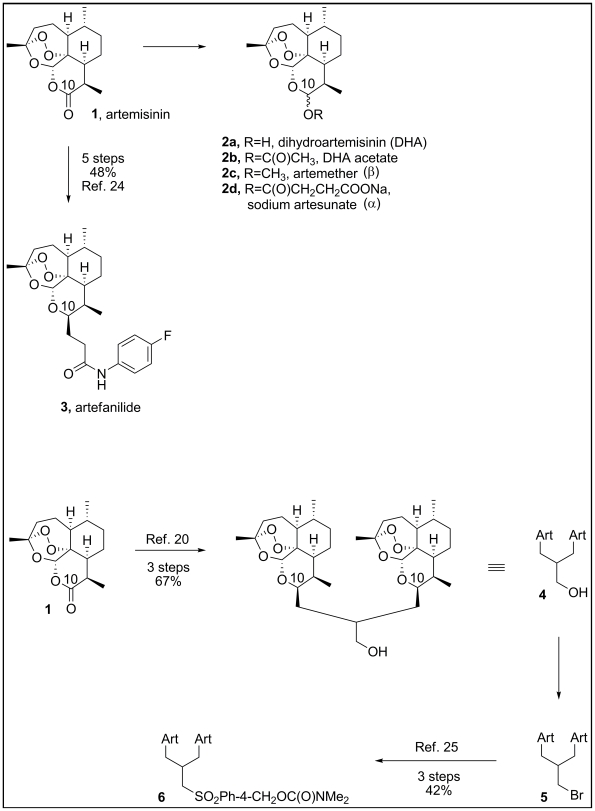
Artemisinin monomers (top) and dimers (bottom) used in this study.

The antimalarial compound, sodium artesunate (Fig. 1, **2d**), a semisynthetic derivative of artemisinin (Fig. 1, **1**), has good tolerability, and lacks significant adverse side effects [16]. In addition to its antimalarial activity, artesunate is cytotoxic to several cancer cell lines [17]. Recently, artesunate was reported to inhibit CMV replication *in-vitro* and in a rat CMV model, exhibiting similar antiviral activity (same micromolar range) to ganciclovir, while demonstrating no cytotoxicity [18], [19]. The *in-vitro* inhibition of clinical isolates ranged from 50–80% using 11.1 µM of artesunate [19]. The parent substance, artemisinin, had lower anti-CMV activity compared to artesunate, suggesting that different artemisinin derivatives may have variable effects on CMV replication.

**Figure 2:**
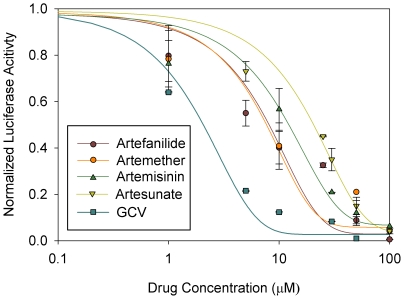
Relative lucifersae activity in CMV-infected HFF treated with four artemisinin monomers. Luciferase assay was performed 72 hr after HFF were treated with compounds and infected with the recombinant luciferase-expressing CMV. Values on Y axis adjusted to 0–1 scale. Mean±SD values are presented.

Dihydroartemisinin (Fig. 1, **2a**, DHA), artemether (**2c**) and artesunate (**2d**) were originally prepared in China in the 1970s. These derivatives and others including artemisone, arteether and artelinic acid are known as monomeric artemisinins. Artemisinin dimers were later synthesized for use as a single dose therapy for malaria in order to improve compliance. These orally active compounds display potent antimalarial and anticancer activities [20], [21]. Other than artemisinin and artesunate, artemisinin monomers and novel artemisinin dimers have not been tested as potential anti-CMV compounds.

We evaluated the *in-vitro* anti-CMV activity of four artemisinin monomers: artemisinin (**1**), artesunate (**2d**), artemether (**2c**), and artefanilide (**3**) and two artemisinin dimers, each incorporating two artemisinin units: dimer primary alcohol (**4**) and dimer sulfone dimethyl carbamate (**6**). We show here that, based on the concentration required for complete inhibition of CMV replication, artemisinin dimers have up to 500 fold higher activity against CMV replication than the artemisinin monomers.

## Materials and Methods

### Ethics Statement

The clinical isolate, SB, was collected at the University of Alabama at Birmingham, after obtaining local approval from the office of Institutional Review Board and written informed consent. The isolate was provided to Johns Hopkins with no identifiers that can link to a specific subject. The Johns Hopkins School of Medicine Office of Human Subject Research Institutional Review Board (IRB-X) determined that the research qualified for an exemption under 45 CFR 46.101(b).

### Cells and viruses

Human Foreskin Fibroblasts (HFF) passage 12–16 and human lung fibroblasts (HEL) passage 8–12 (from ATCC) were maintained in DMEM containing 10% fetal bovine serum and used for infections with the viruses. Cells in concentrations of 5×10^4^ and 1×10^4^ were seeded 24 hours prior to infection on each well of 24-and 96-well plates respectively.

The CMV strains used for infections were the highly-passaged Towne virus, and a clinical isolate obtained from the urine of a neonate with congenital CMV infection (SB) and passaged once in tissue culture. Experiments were performed with multiplicity of infection (MOI) of 0.1, 0.5, 1, and 3. Initial experiments were performed using a recombinant human CMV-green fluorescent protein (GFP) virus, derived from Towne strain. This virus has a 9-kb deletion from the dispensable unique short (US) region from US1 to US12, and instead contains the bacterial artificial chromosome sequences and a GFP expression cassette [22]. Based on pilot experiments using Towne-GFP we elected to use a more sensitive luciferase reporter system to evaluate potential differences in CMV inhibition by various artemisinin derivatives. A recombinant virus expressing luciferase reporter gene under the control of UL99 (pp28) late promoter was generated by insertion of the reporter gene between the US9 and US10 ORFs in the Towne genome. This extragenic reporter gene displays authentic late transcription characteristics after infection of HFF [23]. Pp28 –luciferase expression is strongly activated at 48–72 hours and is almost completely inhibited in the presence of DNA synthesis inhibitors (GCV).

### Compounds

Ganciclovir (GCV) was obtained from Roche, USA and stock was prepared in aqueous solution. Artemisinin was obtained from Sigma-Aldrich. Artesunate (**1**), artemether (**2C**), artefanilde (**3**), dimer primary alcohol (**4**) and dimer sulfone dimethyl carbamate (**6**) were synthesized at Johns Hopkins University (GHP) [24], [25]. Stocks of artemisinin monomers and dimers were prepared in dimethyl sulfoxide (DMSO) and stored in −20°C. Synthetic compounds were at least 98% pure based on high performance liquid chromatography. The DMSO itself was tested in CMV-infected cells and it did not have any anti-viral activity. Fig. 1 depicts the monomers and dimers used in this study.

### Antiviral assays

Towne-GFP, Towne–luciferase, and SB viruses were used in these assays. HFF or HEL were grown to subconfluent monolayers and infected with the CMV viruses at MOI 0.1 to 3. Based on previously reported methods, cells were treated with artemisinin derivatives at various concentrations and infected with CMV 30 min thereafter [19]. The concentration of each compound was calculated and adjusted by volume such that it was constant throughout the experiment. Following 90 minute incubation, media was replaced with fresh media containing the drug used. Infected and treated cells were incubated at 37°C in a 5% CO_2_ atmosphere for 3–10 days depending on the virus and the anti-viral assay. The recombinant luciferase-expressing virus was incubated for 72 hours, the GFP virus was incubated for 5 days, and the clinical isolate for 10 days. All experiments were subsequently repeated with compounds added to wells just after viral inoculation.

For luciferase assay, Wizard® SV Lysis Buffer (Promega, Madison, WI) was added to each well, incubated for 10 minutes at 37°C, followed by 10 minutes of freezing at −80°C and incubation at 37°C for 10 minutes. Luciferase activity was determined in cell extracts using an automated luminescent assay (Promega, Madison, WI). The dynamic range of the luciferase assay is 6–7 logs, and data obtained with it highly correlates with real-time PCR and with plaque reduction assay (manuscript in preparation). Cellular cytotoxicity was determined using CellTiter-Glo® Luminescent Cell Viability Assay (Promega, Madison, WI). The assay determines the number of viable cells in culture based on quantification of the ATP present.

For plaque reduction assay, human embryonic lung cells were seeded into six-well plates and incubated at 37°C one day prior to infection with the recombinant luciferase-expressing CMV. Serial dilutions of GCV, artesunate and artefanilide were used. The virus was diluted to a desired concentration which gave 50–60 plaques per well. Medium was aspirated from the wells, and 0.2 ml of virus suspension was added to each well in triplicates. Plates were incubated for 90 minutes with shaking every 10 min, thereafter drugs were added and a methylcellulose overlay applied to each well. After incubation for 10 days, cells were stained with crystal violet. The stain was aspirated, wells were washed with phosphate-buffered saline, and plaques were counted.

### Western Blot for IE-1 and IE-2

Monoclonal antibodies to CMV immediate early proteins IE-1 and IE-2 (MAb810) and to cellular β-actin were purchased from Millipore (Billerica, MA). Confluent cells were infected with CMV in the presence of artemisinin compounds. At the indicated times cells were harvested in sample buffer (RIPA buffer, Tris 50 mM, Nacl 150 mM, SDS 0.1%, Na Deoxycholate 0.5%, NP40 1%, and protease inhibitor cocktail), boiled and loaded onto SDS-PAGE. Proteins were separated by electrophoresis and transferred to nitrocellulose membrane. After blocking, blots were probed with primary antibody (1∶3,000) overnight at 4°C in phosphate-buffered saline, and Tween. After washing 3 times, blots were probed with HRP-conjugated anti-mouse (Sigma), 1∶5,000 for 1 hour at room temperature. Blots were washed three times in phosphate-buffered saline/Tween, and then developed by enhanced chemiluminescence according to manufacturer protocol.

### CMV US17 real-time PCR assay

To determine the inhibitory effects of artemisinins on DNA copy number of SB clinical CMV isolate, we performed real-time PCR. DNA was isolated from infected cells 10 days post infection using Wizard® SV Genomic DNA Purification System (Promega, Madison, WI). The real-time PCR is based on detection of the highly conserved US17 gene [26]. The primers and probe for US17 are: forward- 5′ GCGTGCTTTTTAGCCTCTGCA-3′, reverse 5′- AAAAGTTTGTGCCCCAACGGTA-3′ and US17 probe FAM- 5′ TGATCGGGCGTTATCGCGTTCT-3′. The limit of detection is 10 copies/reaction (100 copies/mL) and the dynamic range of the assay is 2.4–8.0 log_10_copies/mL.

## Results

Pretreatment of human foreskin fibroblasts (HFF) with different concentrations of artemisinin (**1**) followed by infection with GFP-tagged CMV resulted in dose-dependent reduction in CMV replication (data not shown). To better quantify the extent of reduction and to compare the degree of CMV inhibition by different artemisinin derivatives all subsequent experiments of CMV inhibition were performed using the highly sensitive luciferase assay and the pp28- luciferase expressing CMV.

The four monomers, artemisinin, artesunate, artemether and artefanilide (Fig. 1), were tested first for CMV inhibition in comparison with GCV. These monomers exhibited a similar degree of anti-CMV activity. At 10 µM, ganciclovir (GCV) was more potent in CMV inhibition than all four monomers (Fig. 2). Similar results were obtained by a plaque reduction assay performed in human embryonic lung cells (HEL) with the same luciferase virus; at 10 µM, monomers achieved 40–50% reduction in plaque formation, while GCV reached 80% reduction in plaque formation.

**Figure 3:**
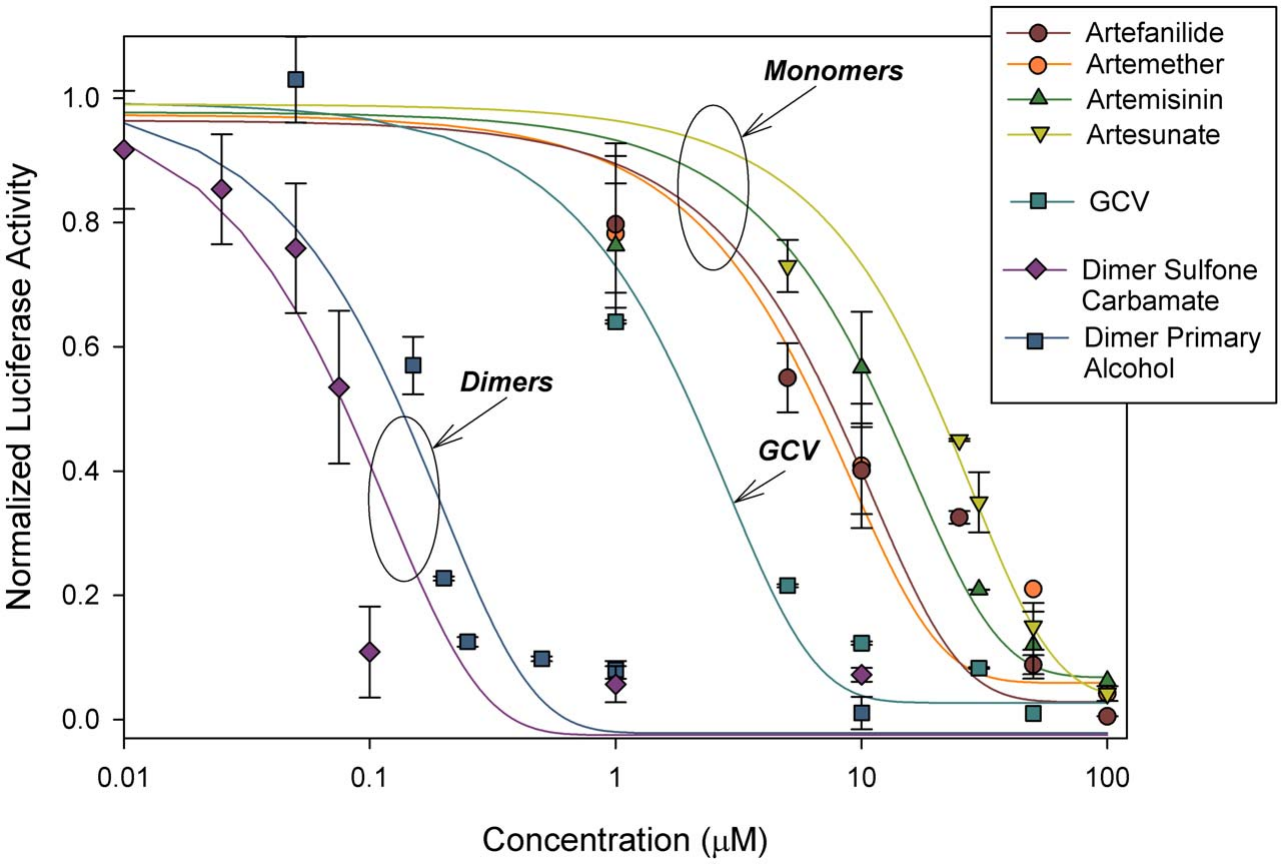
Effect of artemisinin monomers and artemisinin dimers on luciferase expression in CMV-infected HFF. Luciferase assay was performed 72 hr after HFF were treated with monomers or dimers and infected with luciferase-expressing CMV. Values on Y axis adjusted to 0–1 scale. Mean±SD values are presented.

**Figure 4:**
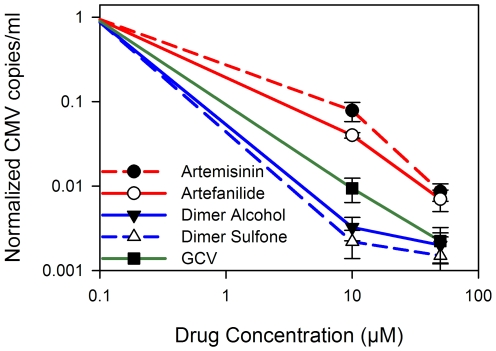
CMV copies/mL at 10 days post infection in the presence of monomers and dimers. HFF were infected with SB clinical isolate (MOI = 1) and treated with artemisinin (**1**), artefanilide (**3**), and two dimers (**4**, and **6**). Ten days following infection DNA was extracted from cells and real-time PCR performed.

We next evaluated CMV inhibition by the four monomers and two dimers. Artemisinin dimers (Fig. 1- **4** and **6**) were significantly more efficient than the monomers in inhibition of pp28-driven luciferase activity (Fig. 3, P<0.0001) without associated cellular cytotoxicity (Table 1). The dimers were up to 500 fold more potent than monomers in achieving complete inhibition of CMV replication. Data presented in the figures was obtained from CMV infected HFF. The same phenomenon of CMV inhibition with dimers compared to monomers was also observed in HEL cells. Error bars presented in figures 2 and 3 represent the standard deviation of 6 experiments, each with three replicates. Normalized luciferase activity is shown as the ratio of luminescence units measured in drug treated CMV-infected cells vs. non-treated CMV-infected cells.

**Table 1:** EC_50_, CC_50_ and selectivity index (SI) for monomers, dimers and GCV.

Compound	CC_50_ (µM)	EC_50_ (µM)	Selectivity index (SI)
Dimer sulfone carbamate (**6**)	28.1±9.6	0.06±0.00	508±173
Dimer primary alcohol (**4**)	57±2.3	0.15±0.02	380±53
GCV	247±33.4	5.6±0.2	44±6.2
Artemisinin (**1**)	72.4±15.7	16.8±4.0	4.3±1.4
Artesunate (**2d**)	77.5±14.4	18.5±5.2	4.2±2.2
Artemether (**2c**)	18.4±7.5	5.3±2.7	3.5±2.2
Artefanilide (**3**)	44.9±3.4	8.1±2.2	5.5±1.6

Dimer primary alcohol (**4**) and dimer sulfone carbamate (**6**) were reproducibly associated with dramatic inhibition of CMV replication using different batches and passages of HFF, and HEL cells.

To confirm that dimers did not block the luciferase enzyme in the recombinant virus, which could have resulted in low luciferase values, whole cell extracts of CMV-infected cells were treated with either dimer primary alcohol or DMSO only. Luciferase activity, measured after 30 minutes of incubation, revealed that luminescence was similar between the dimer-treated (83,200±1,670 units) and the DMSO-treated cells (86,300±2,490), supporting that dimers did not affect the luciferase enzyme itself, but rather inhibited CMV replication.

We evaluated differences in anti-CMV activity between artemisinin monomers and artemisinin dimers using a clinical isolate, SB. Cells were treated with either artemisinin monomers or dimers and DNA was extracted from cell extracts 10 days post infection. Real-time PCR showed that DNA copy number decreased 20–35 fold more with dimers as compared to monomers at 10 µM, and 4 fold more as compared to GCV (Fig. 4).

We calculated the selectivity index (SI) of all artemisinin derivatives and GCV based on the effective drug concentration that results in 50% virus inhibition, EC_50_, and the drug concentration that leads to 50% cellular cytotoxicity, CC_50_ (Table 1). SI was determined as the ratio between CC_50_ and EC_50_. For EC_50_ and CC_50_ reported values represent the mean and SD of data derived from at least five independent experiments performed in triplicates. The concentrations of monomers used for calculating EC_50_ were 1, 5, 10, 25, 50, and 100 µM. The concentrations of dimers used for EC_50_ calculations were 1 nM, 10 nM, 100 nM, 250 nM, 1 µM and 10 µM. The curve fitting toolbox, Matlab software (v7.5), Mathworks (Natick, MA) was used to determine EC_50_ values using a four-parameter logistic regression.

GCV was approximately 10 times more selective than the monomers. The dimer sulfone carbamate (**6**) had the highest selectivity, approximately 10 times more than GCV (Table 1).

To confirm that the difference in anti-CMV activity between monomers and dimers was not a result of instability of the monomers in tissue culture, we determined the anti-toxoplasmosis activity of these compounds in the supernatants of CMV infected cells at 3–5 days post infection. Monomers used in CMV-infected cells proved to inhibit toxoplasmosis in concentrations that correlated with previous reports (Table 2) [27].

**Table 2:** EC_50_ of monomers and dimer primary alcohol for CMV and Toxoplasmosis.

Compound	EC_50_ (µM) - CMV	EC_50_ (µM) - Toxoplasmosis
**Artemisinin**	15.7±4.5	4.1±1.1
**Artesunate**	17.2±3.4	3.2±0.8
**Artemether**	5.1±2.1	0.58±0.22
**Dimer Primary Alcohol**	0.16±0.04	0.40±0.15

**Figure 5:**
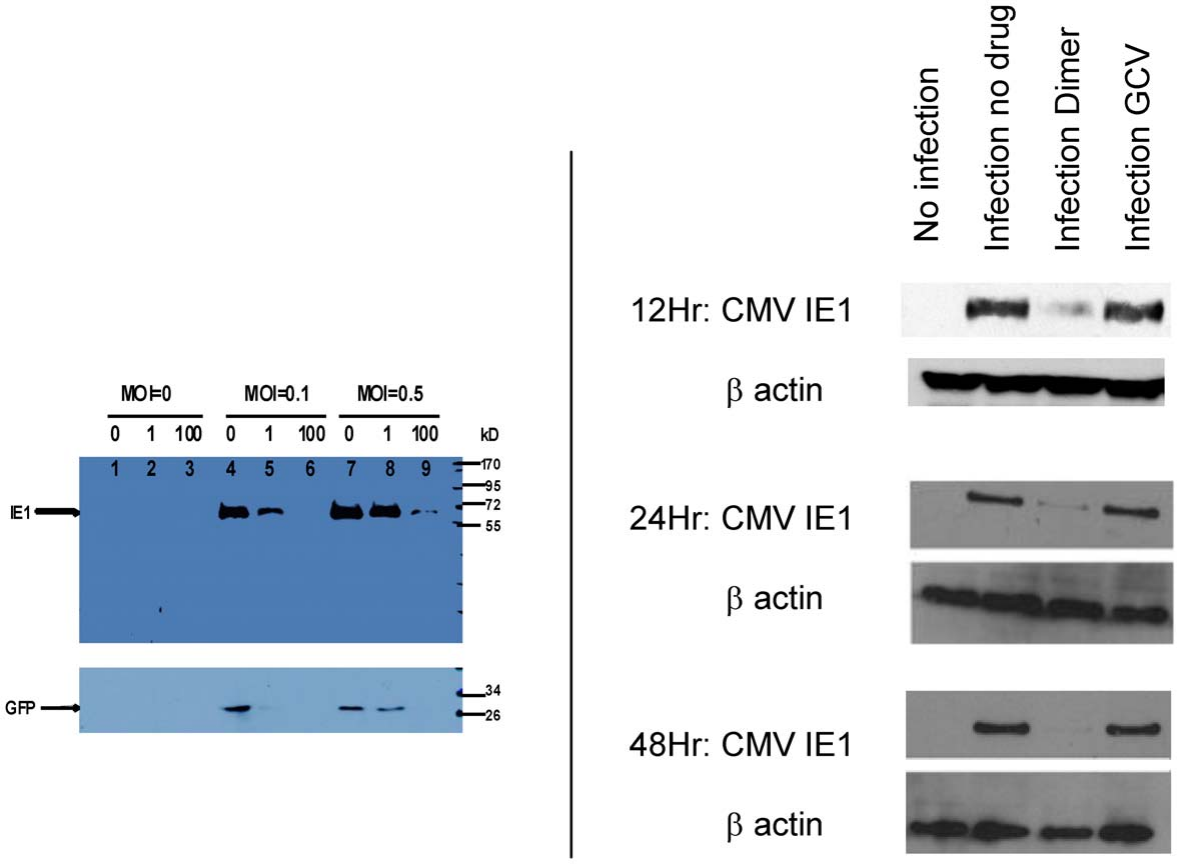
Western blot for IE1, GFP and β actin in artemisinin treated CMV infected cells. 5a: HFF were treated with artemisinin 0, 1 µM and 100 µM and infected with GFP-tagged Towne. Western blot for IE1 and GFP performed at day 5 post infection. 5b: HFF were infected with a clinical isolate, SB, and treated with either dimer primary alcohol (1 µM) or GCV (10 µM). Western blot for IE1 was performed at 12 hr (MOI = 3), 24 hr and 48 hr (MOI = 1) post infection.

The expression of CMV immediate early (IE) protein was tested by western blot to determine whether artemisinins' inhibition of CMV replication occurs early in the virus life cycle. The expression of immediate early 1 (IE1) and GFP in cell lysates treated with artemisinin and infected with Towne-GFP was decreased with 100 µM artemisinin (MOI of 0.5), and undetectable using MOI of 0.1 (Fig. 5a). Further evidence for the early effect of artemisinins on IE1 protein expression was obtained with the clinical isolate (SB, Fig. 5b). IE1 expression was significantly reduced in CMV infected cells (MOI = 1) treated with 1 µM dimer primary alcohol at 24 and 48 hr, but was not affected by 10 µM GCV. The inhibition of IE1 expression with dimer primary alcohol was observed as early as 12 hours post infection (SB, MOI = 3), prior to onset of DNA replication [28].

**Figure 6:**
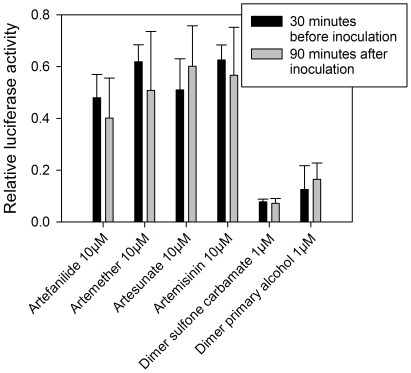
Comparison of CMV inhibition with monomers and dimers before infection and 90 minutes after virus inoculation. Mean±SD values are presented.

To determine whether pretreatment with artemisinin monomers or dimers was necessary to achieve CMV inhibition, all experiments were repeated with infection followed by treatment. HFF were infected with pp28-luciferase expressing CMV (MOI = 1). After 90 minute incubation, unadsorbed virus was removed, and appropriate concentrations of artemisinin monomers or dimers were added. The data obtained with these sets of experiments revealed that pretreatment with the compounds is not required for their anti-CMV activity, and that the exact same degree of CMV inhibition is achieved even when the compounds are added after viral adsorption (Fig. 6).

## Discussion

We report here that artemisinin-derived dimers are potent inhibitors of CMV replication *in-vitro* with no significant cytotoxicity. CMV inhibition by dimers was up to 500 fold higher as compared to the four artemisinin monomers tested- artemisinin, artesunate, artemether and artefanilide. The results obtained were comparable using several anti-viral assays and different viral strains- a laboratory adapted strain (Towne) and a clinical isolate.

CMV is an important pathogen in solid organ transplantation [29], in patients with AIDS [30], and when transmitted during pregnancy from mother to fetus [5]. In addition to these well-documented syndromes, CMV replication has been recently reported in immunocompetent individuals requiring medical care in intensive care units, and in patients with glioblastoma multiforme [7], [9]. Thus, the target population for CMV therapeutics may be growing.

Drugs currently licensed in the US to treat CMV target the viral DNA polymerase and block elongation of the viral DNA chain. They are highly effective in prevention and treatment of CMV disease. However, toxicities, development of drug resistance and inhibition of the host's immune response to CMV are major limitations to their use. New anti-CMV drugs have been developed to overcome these drawbacks. Maribavir, targeting the UL97 kinase [31] is a potent inhibitor of laboratory and clinical isolates of CMV [32]. Despite promising results of phase II multicenter, randomized, double-blind, placebo-controlled study [33], a recent multicenter phase III study in bone marrow transplant recipients showed no statistically significant difference between maribavir and placebo in reducing the rate of CMV disease. In addition to compounds that directly inhibit viral targets, there is a growing interest in compounds that may affect host cell functions required for efficient virus replication [34].

Artesunate (**2d**), a semisynthetic derivative of artemisinin (**1**), the active compound of the Chinese herb *Artemisia annua*, is highly active against malaria parasites. It is available orally, has good tolerability, and lacks significant side effects [16]. Artesunate was first reported to inhibit CMV replication *in-vitro* similar to GCV [19]. A subsequent study performed in a rat CMV model revealed that the parent compound, artemisinin, had lower anti-CMV activity compared to artesunate [18]. In the first report by Efferth [19], artesunate was shown to inhibit several laboratory adapted strains and clinical isolates with the most significant inhibition observed in HEL cells infected with Towne virus. However, in CMV infected HFF inhibition with artesunate was modest. The effect of cell culture conditions on the activity of anti-CMV activity has also been reported for maribavir [35], with more effective suppression of viral growth observed in HEL (lung fibroblasts) than in HFF (foreskin fibroblasts). Our data reveal that in both HFF and HEL cells dimers were significantly more effective in CMV inhibition than monomers.

Artemisinin dimers were originally synthesized to provide a single dose regimen for malaria. Although the first generation of dimers was unstable, the second-generation proved to be thermally and hydrolytically stable [36]. These orally active compounds display potent antimalarial and anticancer activities [20], but they do not have an advantage over artemisinin monomers in clearing malaria parasites as observed in CMV inhibition. Multiple mechanisms may contribute to the anti-cancer activities of artemisinins [37], including inhibition of cell proliferation, induction of G_0_/G_1_ cell cycle arrest and promotion of apoptosis [21]. New dimeric sulfones were reported to cure malaria infected mice with a single oral dose and to be selectively and powerfully cytotoxic to cancer cells [25]. The concentrations effective in cancer cells are similar to those that inhibit CMV replication.

In CMV infected cells it appears that dimers do have an enhanced CMV inhibition over monomers. These inhibitory effects appear early during virus replication cycle as evidenced by decreased expression of CMV IE1 protein, in agreement with prior work [19]. Decreased expression of IE1 will prevent all subsequent steps in the virus replication cycle as shown by decreased DNA synthesis (by real-time PCR) and decreased expression of late CMV proteins (pp28-luciferase assay).

A suggested mechanism of action of artesunate in CMV infection is the inhibition of cellular pathways that play an essential role in viral replication [38]. In artesunate-treated infected cells, Sp1 and NF-kB as well as cellular signaling kinase phosphoinositide 3-kinase (PI3K), required for the activation of Sp1 and NF-κB, were markedly reduced [19]. Although at this time the mechanism of CMV inhibition is largely unknown, better understanding of it will have important clinical implications. Our data suggest that inhibition of CMV replication does not occur at the time of binding of CMV to the cellular receptors, because the compounds are effective even after infection. The fact that dimers are significantly more potent than monomers may suggest improved binding to their specific target than the monomers. Mechanistic studies are ongoing and will be reported in the near future.

In summary, we show here for the first time that artemisinin dimers are dramatically more inhibitory to CMV replication then artemisinin monomers, without associated cytotoxicity. Dimers, although containing only two artemisinin units, are shown here to be much more than twice potent as anti-CMV agents than the corresponding monomeric artemisinins. The anti-viral activity was observed with a laboratory adapted strain and a clinical isolate of CMV. Future work will examine which dimer has the most potent anti-CMV activity and best selectivity index. In addition, other cell types should be tested to confirm the anti-CMV effects of artemisinin monomers and artemisinin dimers. Although experience in humans with artemisinins in CMV disease is not available yet, artesunate was successfully used to treat a child with ganciclovir-resistant CMV following bone marrow transplantation [39].
